# Pharmacokinetics and pharmacodynamics of cyclopropylfentanyl in male rats

**DOI:** 10.1007/s00213-021-05981-x

**Published:** 2021-10-06

**Authors:** Marianne Skov-Skov Bergh, Inger Lise Bogen, Nancy Garibay, Michael H. Baumann

**Affiliations:** 1 Present Address: Section for Drug Abuse Research, Department of Forensic Sciences, Oslo University Hospital, Lovisenberggata 6, 0456 Oslo, Norway; 2 Institute of Basic Medical Sciences, Faculty of Medicine, University of Oslo, Sognsvannsveien 9, 0372 Oslo, Norway; 3 Designer Drug Research Unit, Intramural Research Program, National Institute On Drug Abuse (NIDA), National Institutes of Health (NIH), 333 Cassell Drive, Suite 4400, Baltimore, MD 21224 USA

**Keywords:** Cyclopropylfentanyl, Fentanyl analog, Pharmacokinetics, Pharmacodynamics, Rat, UHPLC-MS/MS

## Abstract

**Background:**

Illicitly manufactured fentanyl and its analogs are a major driving force behind the ongoing opioid crisis. Cyclopropylfentanyl is a fentanyl analog associated with many overdose deaths, but limited knowledge is available about its pharmacology. In the present study, we developed a bioanalytical method for the determination of cyclopropylfentanyl and its main metabolite cyclopropylnorfentanyl and evaluated pharmacokinetic-pharmacodynamic relationships in rats.

**Method:**

An ultra-high performance liquid chromatography tandem mass spectrometry (UHPLC-MS/MS) method was developed and validated for determination of cyclopropylfentanyl and cyclopropylnorfentanyl in rat plasma. Male Sprague–Dawley rats fitted with jugular catheters and temperature transponders received cyclopropylfentanyl (30, 100, and 300 μg/kg) or saline subcutaneously. Blood specimens were withdrawn over an 8-h time period, along with measurements of pharmacodynamic endpoints.

**Results:**

The analytical method was validated, and both analytes exhibited a low limit of quantification (15 pg/mL). Cyclopropylfentanyl caused dose-related increases in hot plate latency (ED_50_ = 48 µg/kg) and catalepsy (ED_50_ = 87 µg/kg) and produced long-lasting hypothermia at the highest dose. Plasma cyclopropylfentanyl rose rapidly in a dose-related fashion, reaching maximal concentration (C_max_) after 15–28 min, whereas metabolite Cmax occurred later at 45–90 min. Cyclopropylfentanyl C_max_ values were similar to concentrations measured in non-fatal intoxications in humans; however, differences in parent drug: metabolite ratio indicated possible interspecies variance in metabolism.

**Conclusion:**

Our study shows that cyclopropylfentanyl produces typical opioid-like effects in male rats. Cyclopropylfentanyl displays much greater analgesic potency when compared to morphine, suggesting that cyclopropylfentanyl poses increased overdose risk for unsuspecting users.

## Introduction

**Fig. 1:**
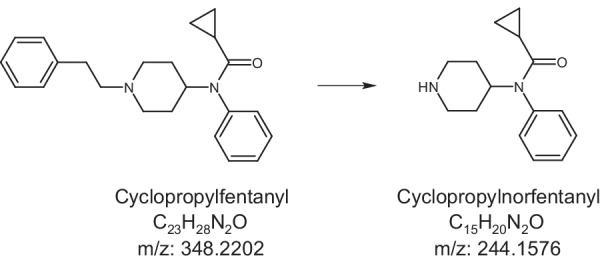
Chemical structures and mass to charge ratios (*m/z*) of cyclopropylfentanyl and cyclopropylnorfentanyl

Since 2013, there has been a surge in the number of overdose deaths related to the synthetic opioid fentanyl and its many analogs (Jannetto et al. 2019; O’Donnell et al. 2020). Cyclopropylfentanyl is a highly potent and dangerous fentanyl analog (see Fig. 1) which has been detected in powders resembling heroin, as well as in liquids and counterfeit prescription drugs (DEA 2017; EMCDDA 2018a). Cyclopropylfentanyl was first described in 1965 in a patent by Paul Janssen (Janssen 1965), but the drug was never approved for therapeutic use (WHO 2018). The compound emerged on the illicit drug market in the United States of America (USA) and in Europe in 2017 (DEA 2017; EMCDDA 2018b) and was temporarily included in Schedule 1 of the Controlled Substances Act by the Drug Enforcement Administration (DEA) in early 2018 (DEA 2018). Cyclopropylfentanyl intoxications produce typical opioid effects such as loss of consciousness, respiratory depression, and miosis (Müller et al. 2019; Wilde et al. 2020). In a recent study from the USA, cyclopropylfentanyl was among the five most commonly detected fentanyl analogs in opioid-related overdose deaths between 2016 and 2018, with several hundred reported cases (O’Donnell et al. 2020). In Europe, cyclopropylfentanyl has been associated with at least 78 fatal intoxications (Sweden, 74; UK, 3; Norway, 1), all occurring in 2017, as well as 144 law enforcement seizures of the drug across six countries (EMCDDA 2018a).

Pharmacokinetic and pharmacodynamic studies of cyclopropylfentanyl are needed to assess the impact of this drug in clinical and forensic toxicology cases. However, information about the *in vivo* biological effects of cyclopropylfentanyl is lacking. *In vitro* studies show that the drug is a fully efficacious µ-opioid receptor agonist with a receptor binding affinity in the low nanomolar range and potency similar to fentanyl (Eshleman et al. 2020; Hassanien et al. 2020; Wilde et al. 2019; Åstrand et al. 2020a; Åstrand et al. 2020b). An animal study performed in the 1960s compared the antinociceptive effects of different fentanyl analogs in mice and rats and reported that cyclopropylfentanyl was slightly less potent than fentanyl but more potent than acetylfentanyl, butyrfentanyl, as well as the reference substance pethidine (Janssen and Van der Eycken 1968).

To our knowledge, no previous studies have examined the* in vivo* pharmacokinetics of cyclopropylfentanyl in humans or animals (EMCDDA 2018a). *In vitro* studies employing human liver hepatocytes and microsomes have identified the *N*-dealkylated metabolite cyclopropylnorfentanyl (Fig. 1) to be the major metabolite of cyclopropylfentanyl (Bergh et al. 2019b; Cutler and Hudson 2019; Wallgren et al. 2020; Åstrand et al. 2018). Cyclopropylnorfentanyl has also been detected in urine and blood samples from forensic toxicology case work (Bergh et al. 2019b; Busardo et al. 2019; Cutler and Hudson 2019; Maher et al. 2018; Matey et al. 2020; Müller et al. 2019; Palaty et al. 2018; Vikingsson et al. 2019; Wallgren et al. 2020; Wilde et al. 2020; Åstrand et al. 2018).

Because of the high potency of cyclopropylfentanyl, the concentrations found in biological samples are low (Roda et al. 2019; Åstrand et al. 2020b). Blood concentrations of cyclopropylfentanyl reported in fatal overdoses were in the range of 0.80–286 ng/mL (Bergh et al. 2018; Brede et al. 2019; Brockbals et al. 2018; Busardo et al. 2019; Danaceau et al. 2020; EMCDDA 2018a; Fagiola et al. 2019; Fogarty et al. 2018; Lee et al. 2019; Maher et al. 2018; Matey et al. 2020), whereas the blood concentrations in non-fatal intoxications were 51 and 76 ng/mL (Müller et al. 2019). To detect such low blood concentrations of drug, highly sensitive analytical methods are required. Several targeted bioanalytical methods determining cyclopropylfentanyl in human blood samples have been developed using liquid chromatography tandem mass spectrometry (LC–MS/MS) (Adamowicz et al. 2020; Bergh et al. 2018; Bergh et al. 2019b; Brockbals et al. 2018; Busardo et al. 2019; Danaceau et al. 2020; Fagiola et al. 2019; Fogarty et al. 2018; Lee et al. 2019; Maher et al. 2018; Matey et al. 2020; Müller et al. 2019; Qin et al. 2019; Sofalvi et al. 2019). However, only a handful of these methods were sensitive enough to successfully detect cyclopropylfentanyl in the sub ng/mL range (< 0.100 ng/mL) (Bergh et al. 2018; Busardo et al. 2019; Danaceau et al. 2020; Qin et al. 2019), and only one method included the major metabolite cyclopropylnorfentanyl (Busardo et al. 2019).

In this study, we evaluated the pharmacodynamic effects and plasma pharmacokinetics of cyclopropylfentanyl in a rat model after subcutaneous (s.c.) injections of cyclopropylfentanyl (30, 100, and 300 μg/kg). For this purpose, an ultra-high performance liquid chromatography (UHPLC)-MS/MS method for determination of cyclopropylfentanyl and cyclopropylnorfentanyl was developed and validated in rat plasma. Adult male rats were fitted with jugular catheters and s.c. temperature transponders, allowing for simultaneous measurements of pharmacodynamic endpoints and serial sampling of blood for analysis of cyclopropylfentanyl and its major metabolite.

## Materials and methods

### Chemicals and reagents

Cyclopropylfentanyl hydrochloride for animal experiments was acquired from Cayman Chemical (Ann Arbor, MI, USA). A stock solution of 1 mg/mL cyclopropylfentanyl in sterile saline was prepared, and 300 µL aliquots were stored at –20 °C. Cyclopropylfentanyl hydrochloride and furanylfentanyl-d_5_ for analytical standards were acquired from Cayman Chemical. Cyclopropylnorfentanyl and norfentanyl-d_5_ were acquired from Chiron AS (Trondheim, Norway). Chromasolv methanol (MeOH) of LC–MS grade was acquired from Honeywell Riedel-de Haën (Seelze, Germany). Ammonium formate and formic acid (98%) were acquired from VWR International AS (Oslo, Norway). Ethyl acetate, n-heptane, nitric acid, and sodium hydroxide were acquired from Merck (Darmstadt, Germany). Disodium tetraborate decahydrate was acquired from Chemi-Teknik AS (Oslo, Norway). Saline was acquired from Hospira (Lake Forest, IL, USA), whereas sodium heparin was acquired from Thomas Scientific (Thomas Scientific, Swedesboro, NJ, USA). Type 1 water (18.2 MΩ) purified with a Synthesis A 10 milli-Q system from Millipore (Billerica, MA, USA) was employed.

### Animals and surgery

Male Sprague–Dawley rats (300–400 g, Envigo, Frederick, MD, USA) were double-housed under conditions of controlled temperature, humidity, and light period (22 ± 2 °C, 45 ± 5% humidity, light period 7 AM–7 PM), with *ad libitum* access to food and water. The animal experiments were approved by the Institutional Animal Care and Use Committee of the NIDA Intramural Research Program, and all procedures were performed in accordance with the National Institutes of Health Guide for the Care and Use of Laboratory Animals. Vivarium facilities were fully accredited by the Association for Assessment and Accreditation of Laboratory Animal Care. Animal experiments were designed to minimize the number of rats included in the study.

Rats were anesthetized with intraperitoneal ketamine (75 mg/kg) and xylazine (5 mg/kg), and indwelling catheters constructed of Silastic (Dow Corning, Midland, MI, USA) and vinyl tubing were implanted into the right jugular vein, as described previously (Concheiro et al. 2014). In short, the proximal Silastic end of the catheter was advanced to the atrium, whereas the distal vinyl end was exteriorized on the nape of the neck and plugged with a metal stylet. Directly after catheter implantation, while still under anesthesia, the rats received surgically implanted temperature transponders (14 × 2 mm, model IPTT-300; Bio Medic Data Systems, Seaford, DE, USA). The transponders were implanted s.c. via a prepackaged sterile guide needle delivery system, along the midline of the back, posterior to the shoulder blades. The radio frequency signals emitted from the temperature transponders were received by a compatible handheld reader system (DAS-7006/7r; Bio Medic Data Systems) to allow noninvasive measurement of body temperature (Elmore and Baumann 2018). Postoperatively, rats were single housed and given one week to recover prior to the experiments. A total of 24 rats were used for these studies, and each subject was used for only one experiment.

### Animal experiments

One week after surgery, rats were brought into the laboratory in their home cages and allowed 1 h to acclimate to the surroundings. Experiments were carried out between 9 AM and 6 PM. Polyethylene extension tubes were attached to 1- mL tuberculin syringes, filled with sterile saline solution, and connected to the vinyl end of the catheters. By threading the extension tubes outside the cage, blood sampling was facilitated by an investigator remote from the rat. Catheters were flushed with 0.3 mL of 48 IU/mL heparin saline to facilitate blood withdrawal.

To prepare cyclopropylfentanyl for injection, a 300 μL aliquot of 1 mg/mL cyclopropylfentanyl was thawed, before being serially diluted in sterile saline solution to yield concentrations of 30, 100, and 300 μg/mL. Groups of rats received s.c. injections of saline vehicle (control) or 30, 100, or 300 μg/kg cyclopropylfentanyl on the lower back between the hips (*N* = 6 rats per dose condition). The drug doses were selected based on preliminary *in vivo* experiments and our published *in vitro* binding data (Baumann et al. 2018), which show that cyclopropylfentanyl and fentanyl display nearly equivalent affinity for µ-opioid receptors. Animals were randomly assigned to each dose group. Blood samples (300 μL) were withdrawn via catheters directly before injection and at 15, 30, 60, 120, 240, and 480 min after injection. Samples were collected into 1 mL tuberculin syringes before being transferred to 1.5 mL plastic tubes containing 5 μL of 250 mM sodium metabisulfite as a preservative and 5 μL of 1000 IU/mL heparin as an anticoagulant. Blood samples were centrifuged for 10 min at 1000 g at 4 °C. Plasma was decanted into cryovials and stored at − 80 °C until analysis. The rats received an equal volume of saline solution (300 µL) via the intravenous catheter after each blood withdrawal to maintain volume and osmotic homeostasis.

Pharmacodynamic endpoints were determined at each blood withdrawal by an experienced rater. Catalepsy score and body temperature were obtained just prior to each blood withdrawal, whereas hot plate latency was measured thereafter. On each test day, one investigator prepared solutions of cyclopropylfentanyl and administered the drug to rats, whereas another investigator performed the behavioral scoring with no knowledge about which dose had been administered. Over the course of the 1-min observation period, catalepsy behaviors were scored based on the following three overt symptoms: immobility, flattened body posture, and splayed limbs, as previously described (Elmore and Baumann 2018). Each symptom was scored as 1 = absent or 2 = present at each time point. For each rat, catalepsy scores at each time point were summed, giving a minimum possible score of 3 and a maximum possible score of 6. Subsequently, body temperature was rapidly measured using a handheld reader sensitive to signals emitted by the surgically implanted transponder. Immediately after each blood withdrawal, rats were placed on a hot plate analgesia meter (IITC Life Sciences, Woodland Hills, CA, USA) set at 52 °C. Rats were removed from the hot plate when they exhibited jumping, flinching, or paw licking as a response to the heat stimulus and were then returned to their home cages. A timer triggered by a foot pedal was used to record the time spent on the hot plate. To prevent tissue damage, a 45 s cut-off was employed.

### Analytical methods

Determination of cyclopropylfentanyl and cyclopropylnorfentanyl in rat plasma samples was performed using a previously described method (Bergh et al. 2018) with minor modifications. Stock solutions of cyclopropylfentanyl, cyclopropylnorfentanyl, and their internal standards (ISs) were prepared in MeOH and stored at − 20 °C. We used furanylfentanyl-d_5_ and norfentanyl-d_5_ as ISs for cyclopropylfentanyl and cyclopropylnorfentanyl, respectively. The optimal choice would have been to use isotopic labeled cyclopropylfentanyl and cyclopropylnorfentanyl, but at the time our method was developed and validated, such compounds were not commercially available. The ISs were therefore chosen based on our previously published and fully validated method developed for determination of cyclopropylfentanyl and 25 other fentanyl analogs (Bergh et al. 2018). Here, furanylfentanyl-d_5_ was used as IS for cyclopropylfentanyl, whereas norfentanyl-d_5_ was used as IS for all N-dealkylated metabolites, with acceptable results. Working solutions for 7 calibrators and 5 quality control (QC) samples were prepared separately by diluting stock solutions in MeOH. Calibrators (10–5000 pg/mL) and QC samples (7.5–4000 pg/mL) were prepared by diluting 25 µL of the working solutions with 100 µL commercial pooled blank Sprague Dawley rat plasma containing K_2_EDTA (Tebu-Bio, Roskilde, Denmark).

Rat plasma samples (100 μL) were transferred to plastic tubes, and IS and MeOH were added prior to vortexing. Borate buffer (pH 11) was added, and the tubes briefly vortexed before addition of an ethyl acetate/heptane mixture, followed by 1 min of vortexing. The tubes were centrifuged, and the supernatants were transferred to glass tubes containing nitric acid in MeOH and evaporated to dryness under a stream of N_2_. The samples were reconstituted in mobile phase and centrifuged prior to transfer of the supernatants to autosampler vials for UHPLC-MS/MS analysis.

Rat plasma samples were analyzed using an Acquity UHPLC™ system (Waters, Milford, MA, USA) coupled to a Xevo-TQS triple quadrupole MS with an electrospray ionization interface (Waters). The analytes were chromatographically separated on a Kinetex biphenyl column (Phenomenex, Verløse, Denmark) kept at 60 °C with a mobile phase consisting of 10 mM ammonium formate pH 3.1 (solvent A) and MeOH (solvent B) delivered at a flow rate of 0.5 mL/min. The separation was performed using a 9-min gradient profile. Data were obtained and processed using Masslynx™ 4.1 software (Waters). For more detailed information about the UHPLC-MS/MS analysis, see Bergh et al. (2018). The MS/MS parameters used, and the retention times of the analytes and ISs, are presented in Table 1.

**Table 1:** MRM transitions, cone voltages, collision energies, and retention times

Analyte	MRM transitions	MS/MS parameters		Retention time (RT)^a^	
		Cone voltage (V)	Collision energy (eV)	RT (min)	CV (%)
CPF	349.2 > 105.1	8	40	5.8	0.5
	**349.2 > 188.2**	8	25		
CPNF	**238.5 > 84.1**	28	16	2.9	0.5
	238.5 > 155.1	28	16		
FF-d_5_	380.2 > 105.0	40	38	5.7	0.6
	**380.2 > 188.1**	40	22		
NF-d_5_	**238.5 > 84.1**	18	16	2.5	0.7
	238.5 > 155.1	18	16		

^a^Transitions used for quantification are written in bold characters

^b^Retention time was calculated based on eight assays of calibrators and QC samples

Abbreviations: *CPF* cyclopropylfentanyl, *CPNF* cyclopropylnorfentanyl, *FF-d*_*5*_ furanylfentanyl-d_5_, *NF-d*_*5*_ Norfentanyl-d_5_

### Method validation

The UHPLC-MS/MS method was validated in accordance with the Scientific Working Group for Forensic Toxicology guidelines (AAFS 2019), with minor modifications. The validation was performed by examining the following parameters: linearity, intermediate precision and bias, limit of detection (LOD), limit of quantification (LOQ), recovery, matrix effects (ME), matrix interferences, stability, and carry-over.

The linearity was determined based on 8 assays of 7 calibrators prepared in pooled rat plasma from drug-naïve control rats with one replicate per concentration level. Weighted calibration curves (1/x), excluding the origin, were constructed by plotting calibrator concentration against analyte/IS peak height ratio. The calibration curves were considered acceptable with a correlation coefficient (*R*^2^) ≥ 0.99 and residuals ≤  ± 20%.

Intermediate precision and accuracy were determined based on 8 assays of 4 different QC sample concentrations prepared in both pooled plasma from drug-naïve control rats and in commercial pooled rat plasma with one parallel pr. matrix source. Precision was determined as the coefficient of variation (% CV). Accuracy, given as bias, was calculated as the percent deviation between the measured mean of the different QC samples and the respective nominal concentration. Intermediate precision and accuracy were determined for all assays collectively and deemed acceptable at a % CV and bias ≤  ± 20%.

LOD was determined based on three assays of five different QC sample concentrations prepared in both pooled rat plasma from drug-naïve control rats and commercial pooled rat plasma with one parallel pr. matrix source. LOD was defined as the lowest concentration which produced a signal-to-noise ratio (S/N) ≥ 3. LOQ was determined based on 8 assays of 4 different QC sample concentrations prepared in both pooled plasma from drug-naïve control rats and commercial pooled rat plasma with one parallel pr. matrix source. LOQ was defined as the lowest QC sample concentration where the S/N ≥ 10 and the intermediate precision and accuracy were ≤  ± 20% for the transition of quantification.

Recovery and ME were determined by analyzing three sets of samples fortified with analytes at two concentration levels (25 and 4000 pg/mL). Ten blank plasma samples from two different sources (4 samples of pooled plasma from drug-naïve control rats and 6 samples of commercial pooled rat plasma) were fortified with analyte pre-extraction (set 1) or post-extraction (set 2). Additionally, five replicates of reconstitution solution were fortified with analyte (set 3). IS was added to all samples post-extraction. Recovery was determined as the ratio between the peak heights of analyte added pre-extraction (set 1) and the peak height of analyte added post-extraction (set 2). ME was determined as the ratio between the peak height of analyte added post-extraction (set 2) and the peak height of analyte added to reconstitution solution (set 3), as described by Matuszewski et al. (2003). The ME was deemed acceptable within the range of 80–120%.

Matrix interferences were assessed in the plasma samples taken from the rats prior to cyclopropylfentanyl exposure (N = 17). Because neither of the ISs used were isotope labeled analogs of the analytes, which can contain traces of the unlabeled analyte, interferences from the ISs were not evaluated.

The stability of cyclopropylfentanyl and cyclopropylnorfentanyl in fortified commercial pooled rat plasma and extracted rat plasma samples fortified pre-extraction were evaluated in triplicates at two concentration levels (25 and 4000 pg/mL). The stability of fortified plasma samples was investigated after two freeze/thaw cycles and after storage for up to 2 months at − 80 °C. The stability of extracted samples was evaluated for up to 2 days in the autosampler at 10 °C. Samples were deemed stable if the deviation from the initial concentration was ≤  ± 20%.

Carry-over was assessed by injecting an extracted rat plasma sample fortified with the highest calibrator (5000 pg/mL) succeeded by three samples of blank extracted matrix. Carry-over was considered present if the blank samples displayed a peak height > 10% of the peak height at LOQ.

### Data analysis and statistics

All pharmacodynamic and pharmacokinetic data were statistically evaluated employing GraphPad Prism version 8.0 (GraphPad Software, La Jolla, CA, USA). *ED*_50_ values were calculated in GraphPad Prism by nonlinear regression analyses of mean catalepsy scores and hot plate responses over the first 2 h after cyclopropylfentanyl injection. Time course data for body temperature, catalepsy scores, and hot plate latency were evaluated using two-way analysis of variance (dose × time) followed by Tukey’s multiple comparison tests. Time-concentration profiles for cyclopropylfentanyl and cyclopropylnorfentanyl were subjected to two-way analysis of variance (dose × time) followed by Tukey’s multiple comparison tests. Thermo Kinetica version 5.1 (Thermo Fisher Scientific, Philadelphia, PA, USA) was used to determine pharmacokinetic constants for cyclopropylfentanyl and its metabolite cyclopropylnorfentanyl, including concentration maximum (*C*_max_), time for concentration maximum (*T*_max_), area-under-the-curve (*AUC*), elimination constant (K_e_), and plasma half-life (*T*_1/2_). To determine differences between dose groups, pharmacokinetic constants for each analyte were compared by one-way analysis of variance (dose) followed by Tukey’s post-hoc test. The observed *AUC* values, from 0 to 8 h post-injection, were compared to predicted *AUC* values which were calculated for the 100 and 300 μg/kg cyclopropylfentanyl doses by multiplying the observed value at 30 μg/kg by 3.33 and 10, respectively. Predicted and observed values for cyclopropylfentanyl and cyclopropylnorfentanyl *AUC* were analyzed by two-way ANOVA (dose × condition). Relationships between plasma concentrations of analytes and body temperature, catalepsy score, or hot plate latency were evaluated using a Pearson’s correlation analysis. Specifically, for each subject, the *AUC* value calculated for each analyte was plotted with respect to the mean temperature, summed catalepsy score, or mean hot plate latency values across all times points. *p* < 0.05 was employed as the minimum threshold for statistical significance for all comparisons performed.

## Results

### Method validation

**Fig. 2:**
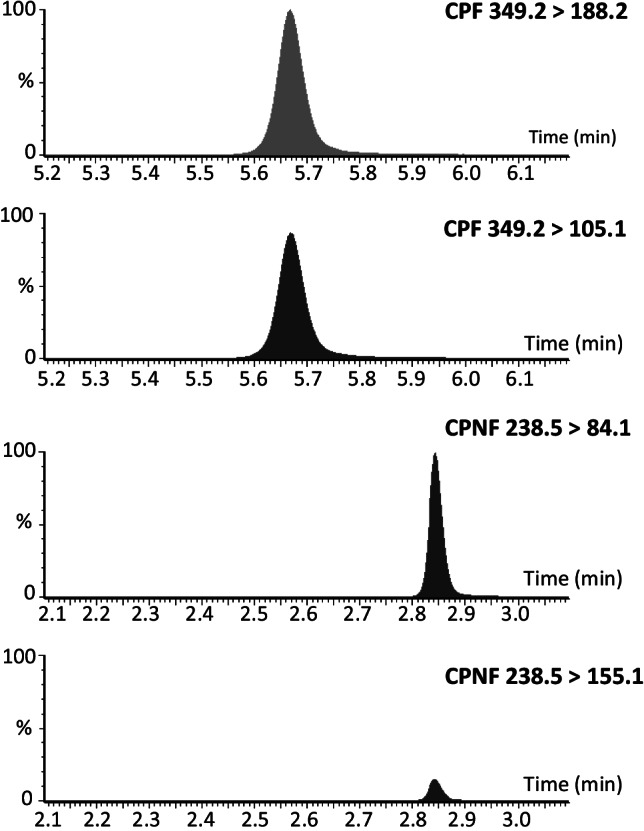
MRM chromatograms of a rat plasma sample obtained 4 h after injection of 300 µg/kg cyclopropylfentanyl analyzed with the presented UHPLC-MS/MS method. Abbreviations: *CPF*, cyclopropylfentanyl; *CPNF*, cyclopropylnorfentanyl

Figure 2 displays representative MRM chromatograms for cyclopropylfentanyl and cyclopropylnorfentanyl in rat plasma samples analyzed with the presented UHPLC-MS/MS method. The retention times for cyclopropylfentanyl and cyclopropylnorfentanyl were 5.8 min and 2.9 min, respectively. The LOD for cyclopropylfentanyl and cyclopropylnorfentanyl was 7.5 pg/mL, while the LOQ for the compounds was 15 pg/mL. The calibration curves for both analytes were linear within the range of 10–5000 pg/mL with correlation coefficients (*R*^2^) ≥ 0.99 and residuals ≤  ± 20%.

The validation data for cyclopropylfentanyl and cyclopropylnorfentanyl are listed in Tables 2 and 3. The intermediate precision and accuracy for cyclopropylfentanyl and cyclopropylnorfentanyl were acceptable for all four QC sample concentrations (15, 25, 500, and 4000 pg/mL) with a % CV ≤ 7.6% and bias ≤ 4.5%. No matrix interferences were observed for cyclopropylfentanyl in the plasma samples. In the cyclopropylnorfentanyl chromatograms, a peak was observed eluting immediately before cyclopropylnorfentanyl. However, the peak had close to baseline separation with cyclopropylnorfentanyl, and was therefore not likely to interfere with analyte quantification. Recovery was high for cyclopropylfentanyl (93–94%) and cyclopropylnorfentanyl (85–88%), and the four analytes did not display ME (98–100%). Carry-over was not observed for either compound. Stability studies were carried out in fortified rat plasma samples and extracted plasma samples fortified pre-extraction (Table 3). Cyclopropylfentanyl and cyclopropylnorfentanyl were stable in extracted plasma samples stored in the autosampler (10 °C) for 48 h. Both analytes were stable in fortified plasma for two freeze/thaw cycles and for up to 2 months of storage at − 80 °C.

**Table 2:** Intermediate precision and accuracy, matrix effects, and recovery

Analyte	Nominal concentration	Intermediate precision and accuracy^a^		Matrix effects (ME)^b^			Recovery	
	(pg/mL)	CV (%)	Bias (%)	ME (%)	CV (%)	ME corr.^c^ (%)	%	CV (%)
CPF	15	7.3	− 2.5					
	25	7.6	− 1.4	100	3.7	107	93	6.0
	501	6.6	− 2.9					
	4008	5.1	1.0	100	2.2	100	94	5.9
CPNF	15	4.4	4.5					
	25	5.3	3.0	99	5.3	101	88	6.0
	500	6.7	− 1.9					
	4008	6.0	− 4.4	98	1.8	97	85	6.2

^a^Intermediate precision and accuracy were acceptable at % CV and bias ≤  ± 20%

^b^ME was acceptable within the range of 80–120%

^c^ME corrected with IS

Abbreviations: *CPF* cyclopropylfentanyl, *CPNF* cyclopropylnorfentanyl

**Table 3:** Stability data for cyclopropylfentanyl and cyclopropylnorfentanyl in extracted samples stored at 10 °C and in plasma stored at − 80 °C

Analyte	Nominal concentration (pg/mL)	Stability (%) of extracted samples stored at 10 °C ^a,b^		Stability (%)^a,b^ of plasma samples stored at − 80 °C			
		24 h	48 h	24 h	1 month	2 months	2 freeze–thaw cycles
CPF	25	− 2.1 (10)	− 3.9 (2.2)	− 9.6 (8.2)	− 17 (4.8)	− 0.5 (3.1)	− 12 (6.3)
	4008	− 1.1 (3.4)	− 0.2 (3.8)	− 4.7 (6.2)	− 17 (6.9)	− 16 (11)	8.4 (3.6)
CPNF	25	− 3.0 (8.6)	1.6 (3.9)	− 3.1 (0.8)	− 3.0 (4.0)	− 5.7 (3.2)	− 3.0 (7.3)
	4008	− 5.5 (1.5)	0.2 (3.1)	− 9.8 (3.1)	− 13 (3.3)	− 19 (9.5)	4.5 (2.1)

^a^% CV in parentheses

^b^Samples were considered stable if the deviation from the initial concentration was ≤  ± 20%

Abbreviations: *CPF* cyclopropylfentanyl, *CPNF* cyclopropylnorfentanyl

### Pharmacodynamic effects

**Fig. 3:**
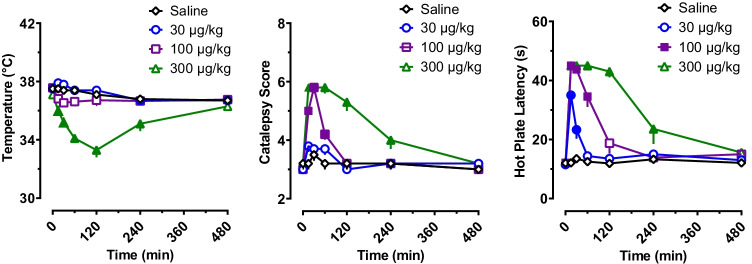
Time-course of pharmacodynamic effects induced by s.c. cyclopropylfentanyl administration (30, 100, and 300 µg/kg) in male rats. Body temperature, catalepsy score, and hot plate latency were measured at 0, 15, 30, 60, 120, 240, and 480 min after injection. Control animals received s.c. saline vehicle (1 mL/kg). Data are expressed as the mean ± SEM for N = 6 rats/group. Filled symbols represent significant differences when compared to saline-treated animals at a given time point (Tukey’s multiple comparison test, *p* < 0.05)

Figure 3 depicts the time-course effects of s.c. cyclopropylfentanyl administration (30, 100, and 300 µg/kg) on core body temperature, catalepsy, and hot plate latency in male rats. Body temperature was significantly affected by dose (*F* (3, 133) = 101.30, *p* < 0.0001) and time (*F* (6, 133) = 12.71, *p* < 0.0001), with a significant dose × time interaction (*F* (18, 133) = 7.385, *p* < 0.0001). Cyclopropylfentanyl induced a dose-related decrease in temperature when compared to saline-treated controls. No significant changes in core temperature were found after injection of 30 and 100 μg/kg, but a significant hypothermic response was observed after 300 μg/kg reaching a nadir of 3.8 °C at 2 h post-injection. Temperature measures returned to control values by 8 h after 300 μg/kg cyclopropylfentanyl.

Catalepsy score was significantly affected by cyclopropylfentanyl dose (*F* (3, 133) = 90.84, *p* < 0.0001) and time (*F* (18, 133) = 10.47, *p* < 0.0001), with a significant dose × time interaction (*F* (6, 133) = 45.35, *p* < 0.0001). Cyclopropylfentanyl produced a dose-related increase in catalepsy score when compared to saline-treated controls, which was characterized by immobility, flattened body posture, and splayed hind limbs. Other effects of the drug included exophthalmos and shallow labored breathing, but these endpoints were not systematically evaluated. The 30 μg/kg dose of cyclopropylfentanyl did not induce catalepsy, whereas the 100 and 300 µg/kg doses increased catalepsy lasting for 1 h and 4 h post-injection, respectively. The rats exhibited the highest catalepsy scores at 15–60 min post-injection. Nonlinear regression of the mean catalepsy scores over the first 2 h post-injection revealed an *ED*_50_ of 87 µg/kg.

Hot plate latency was significantly affected by cyclopropylfentanyl dose (*F* (3, 133) = 141.80, *p* < 0.0001) and time (*F* (6, 133) = 74.41, *p* < 0.0001), with a significant dose × time interaction (*F* (18, 133) = 16.83, *p* < 0.0001). Cyclopropylfentanyl produced a dose-related increase in hot plate latency when compared to saline-treated controls, which indicates an analgesic effect. Post hoc tests revealed that the lowest dose of cyclopropylfentanyl (30 µg/kg) significantly increased hot plate latency for 30 min post-injection, but the maximum latency cut-off of 45 s was not reached with this dose. Animals that received 100 μg/kg and 300 µg/kg cyclopropylfentanyl exhibited the maximum cut-off latency time of 45 s by 15 min after injection, and the latency was increased for 1 h and 4 h post-injection, respectively. Nonlinear regression of the mean hot plate responses over the first 2 h post-injection revealed an *ED*_50_ of 48 µg/kg.

### Plasma pharmacokinetics

**Fig. 4:**
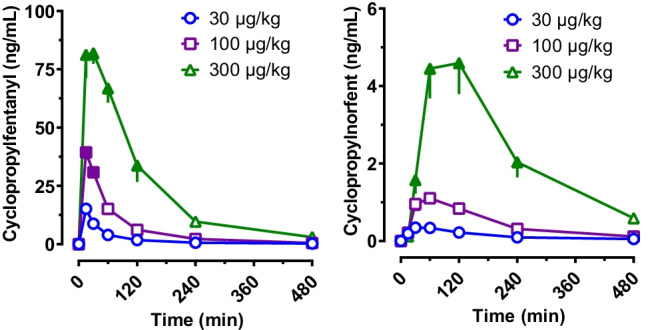
Concentration–time profiles for cyclopropylfentanyl and cyclopropylnorfentanyl in rats after s.c. administration of 30, 100, or 300 µg/kg cyclopropylfentanyl. Rats fitted with indwelling jugular catheters received cyclopropylfentanyl at time zero. Blood samples were withdrawn via the catheters immediately prior to and 15, 30, 60, 120, 240, and 480 min after cyclopropylfentanyl injection. Plasma samples were assayed for analytes using UHPLC-MS/MS. Data are expressed as the mean ± SEM for *N* = 6 rats/group. Filled symbols represent significant differences when compared to the low-dose group (30 µg/kg) at a given time point (Tukey’s multiple comparison test, *p* < 0.05)

Figure 4 depicts the time-concentration profiles for plasma cyclopropylfentanyl and cyclopropylnorfentanyl after s.c. injection of cyclopropylfentanyl to male rats. The time-concentration profiles for cyclopropylfentanyl were significantly affected by dose *F* (2, 98) = 202.80, *p* < 0.0001) and time (*F* (6, 98) = 89.35, *p* < 0.0001), with plasma concentrations rising with increasing dose. Post hoc tests revealed that plasma concentrations of cyclopropylfentanyl were significantly higher after 100 μg/kg and 300 µg/kg when compared to 30 μg/kg for the first 30 min and 2 h after injection, respectively. Pharmacokinetic constants are listed in Table 4. The *C*_max_ after cyclopropylfentanyl injection was significantly altered by the dose administered (*F* (2, 14) = 82.65, *p* < 0.0001), as was *AUC* (*F* (2, 14) = 60.41, *p* < 0.001). Cyclopropylfentanyl *T*_max_ occurred rapidly at 15 min post-injection for the 30 and 100 µg/kg doses but was somewhat delayed for the 300 µg/kg dose, with an average of 27.5 min post-injection. The *T*_1/2_ values for cyclopropylfentanyl were in the range of 89 to 115 min. Post hoc tests showed that *C*_max_ values after administration of 100 µg/kg dose were significantly higher than those observed at the 30 µg/kg dose, while the values after the 300 μg/kg dose were significantly greater than those observed after 30 and 100 μg/kg doses. The *AUC* after administration of 300 μg/kg cyclopropylfentanyl was significantly greater than the *AUC* values found after administration of doses of 30 and 100 µg/kg.

**Table 4:** Pharmacokinetic constants for cyclopropylfentanyl and cyclopropylnorfentanyl after s.c. administration of 30, 100, or 300 µg/kg cyclopropylfentanyl in male rats

Analyte	Drug dose (µg/kg)	*C*_max_ (ng/mL)	*T*_max_ (min)	*AUC*_0-8 h_(min*ng/mL)	*K*_e_	*t*_1/2_ (min)
CPF	30 (*N* = 6)	15.13 ± 1.6	15 ± 0	892.1 ± 52	0.0063 ± 0.0006	115 ± 11
	100 (*N* = 5)	39.40 ± 3.7^a^	15 ± 0	2879 ± 96	0.0079 ± 0.0004	89.1 ± 4.2
	300 (*N* = 6)	90.83 ± 6.2^a,b^	27.5 ± 7.2	10,983 ± 1140^a,b^	0.0074 ± 0.0004	94.5 ± 4.7
CPNF	30 (*N* = 6)	0.361 ± 0.02	45 ± 6.7	77.1 ± 5.7	0.0046 ± 0.0004	160 ± 19
	100 (*N* = 5)	1.18 ± 0.10	54 ± 6.0	232.4 ± 19	0.0054 ± 0.0002	130 ± 5.6
	300 (*N* = 6)	5.57 ± 0.49^a,b^	90 ± 13^a,b^	1127 ± 171^a,b^	0.0059 ± 0.0004^a^	119 ± 7.2

Data are mean ± SEM

^a^Significant difference compared to 30 µg/kg dose (Tukey’s *p* < 0.05)

^b^Significant difference compared to 100 µg/kg dose (Tukey’s, *p* < 0.05)

Abbreviations: *CPF* cyclopropylfentanyl, *CPNF* cyclopropylnorfentanyl, *C*_*max*_ maximal concentration, *T*_*max*_ time for concentration maximum, *AUC*_*0-8 h*_ area-under-the-curve from 0–8 h, *K*_*e*_ elimination rate constant, *t*_*1/2*_ half-life

The time-concentration profiles for plasma cyclopropylnorfentanyl were also significantly affected by cyclopropylfentanyl dose (*F* (2, 98) = 73.31, *p* < 0.0001) and time post-injection (*F* (6, 98) = 22.65, *p* < 0.0001), with concentrations rising with increasing dose (Fig. 4). The plasma concentrations of cyclopropylnorfentanyl were significantly higher after 300 μg/kg cyclopropylfentanyl when compared to those observed after 30 and 100 μg/kg from 30 min to 4 h post-injection. Pharmacokinetic constants for cyclopropylnorfentanyl are listed in Table 4. Cyclopropylnorfentanyl *C*_max_ was significantly altered by the dose administered (*F* (2, 14) = 87.49, *p* < 0.0001), as was *AUC* (*F* (2, 14) = 29.74, *p* < 0.001). Cyclopropylnorfentanyl *T*_max_ occurred between 45 and 90 min and was affected by the dose administered (*F* (2, 14) = 6.59, *p* < 0.01). The *T*_1/2_ values for cyclopropylnorfentanyl were in the range of 119 to 160 min. Post hoc tests showed that *C*_max_, *AUC*, and *T*_max_ values of cyclopropylnorfentanyl after injection of 300 μg/kg cyclopropylfentanyl were significantly greater than those observed after administration of 30 and 100 μg/kg doses.

**Fig. 5:**
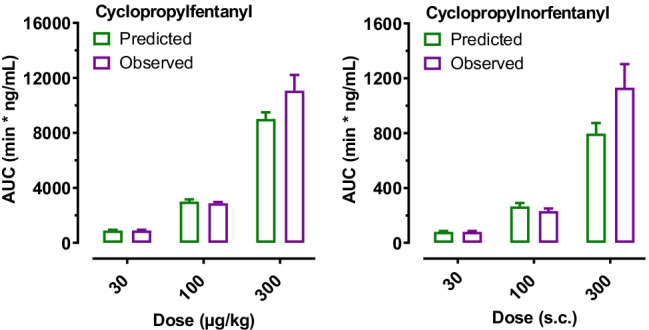
Comparison of observed versus predicted area-under-the-curve (*AUC*) values for cyclopropylfentanyl after s.c. administration of 30, 100, or 300 µg/kg cyclopropylfentanyl in rats. Observed *AUC* values were obtained from time-concentration profiles depicted in Fig. 4, whereas predicted values at 100 and 300 μg/kg doses were calculated by multiplying the observed *AUC* values at 30 μg/kg by 3.33 and 10, respectively. Data are expressed as the mean ± SEM for *N* = 6 rats/group

To examine the possibility of nonlinear pharmacokinetics for cyclopropylfentanyl and cyclopropylnorfentanyl, we compared the observed *AUC* values at 100 and 300 μg/kg to the predicted values, which were determined by multiplying the observed values at 30 μg/kg by a factor of 3.33 and 10, respectively. Two-way ANOVA demonstrated that observed versus predicted *AUC* values for cyclopropylfentanyl and cyclopropylnorfentanyl did not differ significantly (Fig. 5).

### Correlation analyses

**Fig. 6:**
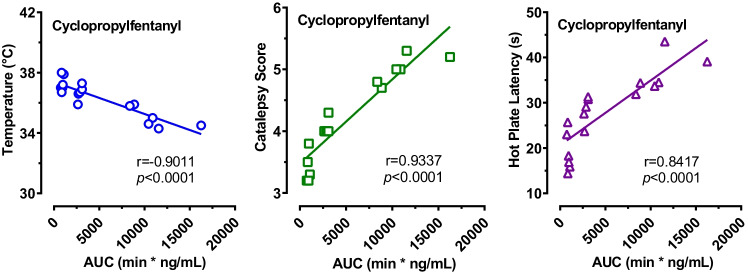
Correlations between *AUC* values for cyclopropylfentanyl versus body temperatures, catalepsy scores, and hot plate latency. Raw data from Figs. 3 and 4 were used to construct the correlation matrices which plot mean temperature, summed catalepsy scores, and mean hot plate latency for each rat versus corresponding *AUC* values for cyclopropylfentanyl in the same subjects over the 8-h session. Pearson’s *r* and *p* values are shown

Since the pharmacokinetic and pharmacodynamic data were measured from the same animals, correlation analyses were performed to evaluate relationships between the plasma concentrations of cyclopropylfentanyl and cyclopropylnorfentanyl and pharmacodynamic endpoints such as temperature, catalepsy, and hot plate latency. This was performed by Pearson correlation analyses, plotting the mean core temperature, catalepsy scores, or hot plate latency values measured during the 8-h experiment for each rat against the *AUC* values from the same subject. Figure 6 shows that plasma cyclopropylfentanyl concentrations were significantly correlated with body temperature, catalepsy scores, and hot plate latency. Core body temperature was negatively correlated with cyclopropylfentanyl *AUC* (*r* =  − 0.9011, *p* < 0.0001), whereas catalepsy (*r* = 0.9337, *p* < 0.0001) and hot plate latency (*r* = 0.8417, *p* < 0.0001) were positively correlated with cyclopropylfentanyl concentrations. Similar findings were observed when relating cyclopropylnorfentanyl *AUC* values to body temperature (*r* =  − 0.9129, *p* < 0.0001), catalepsy scores (*r* = 0.9018, *p* < 0.0001), and hot plate latency (*r* = 0.8253, *p* < 0.0001).

## Discussion

The presence of fentanyl and its various analogs on the illicit drug market has been a major driving force behind the ongoing global opioid overdose epidemic (Jannetto et al. 2019; O’Donnell et al. 2020). Cyclopropylfentanyl is a recently encountered clandestine fentanyl analog associated with a high number of overdose deaths (EMCDDA 2018a; O’Donnell et al. 2020), but limited data is available about its pharmacokinetics and pharmacodynamic effects. In this study, we developed a sensitive bioanalytical method for the determination of cyclopropylfentanyl and its main metabolite cyclopropylnorfentanyl and examined pharmacokinetic-pharmacodynamic relationships in male rats. We show that cyclopropylfentanyl induces a dose-related increase in catalepsy and hot plate latency, displaying an analgesic potency similar to the reported analgesic potency of fentanyl (van den Hoogen and Colpaert 1987; Calcagnetti et al. 1990; Paronis and Holtzman 1992; Megens et al. 1998). Furthermore, the drug produces hypothermia lasting for hours at the highest dose (300 µg/kg). Pharmacodynamic endpoints were found to correlate with plasma concentrations of cyclopropylfentanyl and the metabolite. The ratio between the parent drug and metabolite in our study deviated from the findings from human post-mortem blood samples (Busardo et al. 2019), indicating possible interspecies differences in metabolism of the drug.

There is scant information available regarding the* in vivo* pharmacodynamics of cyclopropylfentanyl (EMCDDA 2018a). *In vitro* studies show that cyclopropylfentanyl displays a binding affinity in the low nanomolar, or sub-nanomolar, range for µ-opioid receptors (Baumann et al. 2018; Eshleman et al. 2020; Hassanien et al. 2020; Wilde et al. 2019; Åstrand et al. 2020a; Åstrand et al. 2020b). A preclinical study from the 1960s compared the antinociceptive effects of various fentanyl analogs and reported that cyclopropylfentanyl exhibits a potency somewhat lower than fentanyl but higher than acetylfentanyl and butyrfentanyl (Janssen and Van der Eycken 1968). In the present work, we demonstrate that cyclopropylfentanyl induces significantly increased hot plate latency at doses from 30 to 300 μg/kg, with an *ED*_50_ of 48 µg/kg. One limitation of our study is that we did not carry out a direct comparison of the *in vivo* pharmacodynamic effects of cyclopropylfentanyl with those of other opioids like morphine or fentanyl. Nonetheless, previous investigations using the rat hot plate test, similar to the methods used here (i.e., 52 °C with a 45-s cutoff), show that *ED*_50_s for s.c. morphine ranged from 2.8 to 6.7 mg/kg (Gunn et al. 2011; Morgan et al. 2006). In previous studies using the rat tail flick test to measure analgesia, the *ED*_50_s for s.c. morphine ranged from 3.3 to 7.1 mg/kg, whereas the *ED*_50_s for fentanyl ranged from 32.0 to 44.0 µg/kg (van den Hoogen and Colpaert 1987; Calcagnetti et al. 1990; Paronis and Holtzman 1992; Megens et al. 1998**)**. Thus, our data suggest that the analgesic potency of cyclopropylfentanyl in the rat (i.e., 48 µg/kg) is in the same dose range as the published analgesic potency of fentanyl, which is about 80–100 times more potent than morphine (Mounteney et al. 2015; UNODC 2017; Varshneya et al. 2019).

Exposure to µ-opioid receptor agonists such as morphine and fentanyl induces catalepsy in rats which is characterized by muscle rigidity and immobility (Chen et al. 1996; Ling and Pasternak 1982; Pasternak et al. 1983). By using a behavior scoring method based on immobility, flattened body posture, and splayed limbs, we demonstrate that cyclopropylfentanyl produces catalepsy-like effects at 100 and 300 µg/kg doses, lasting up to 4 h after the higher dose. The *ED*_50_ for cyclopropylfentanyl to induce catalepsy was right shifted when compared to that of analgesia (i.e., 87 µg/kg), which agrees with the findings from previous studies reporting that higher morphine doses are needed to induce cataleptic effects compared to analgesic effects (Pöyhiä and Kalso 1992; Taracha et al. 2009). We also show that cyclopropylfentanyl induces hypothermia at the highest dose administered (300 μg/kg), with temperatures decreasing in a manner similar to the effects of other synthetic opioids such as carfentanil and U-47700 (Bergh et al. 2019a; Truver et al. 2020). Prior work demonstrates that the effects of opioids on body temperature are dose-specific, with hyperthermia at low doses and hypothermia at high doses (Geller et al. 1983; Rawls and Benamar 2011). Geller et al. (1983) found that s.c. administration of 32 mg/kg morphine or 200 µg/kg fentanyl decreased core body temperature in male Sprague–Dawley rats by about 1.5 °C, while we observed > 3.0 °C drop in temperature after s.c. administration of 300 μg/kg cyclopropylfentanyl. Altogether, the results suggest that cyclopropylfentanyl induces hypothermic effects in the rat in the same dose range reported for fentanyl, which is approximately 100-fold more potent than morphine in this regard. Future investigations are warranted to examine other biological effects produced by cyclopropylfentanyl in rodent models, including rewarding effects, tolerance, dependence, and respiratory depression.

In the present study, we validated a UHPLC-MS/MS method that allows for determination of cyclopropylfentanyl and cyclopropylnorfentanyl in small-volume rat plasma samples, even after administration of low cyclopropylfentanyl doses (30 μg/kg). To the best of our knowledge, this is the first reported UHPLC-MS/MS method established for this purpose. The method displayed an LOQ of 15 pg/mL for cyclopropylfentanyl and cyclopropylnorfentanyl, meaning a sensitivity comparable (Bergh et al. 2018; Busardo et al. 2019) or superior to previously reported LC–MS/MS methods for this fentanyl analog (Brockbals et al. 2018; Danaceau et al. 2020; Fagiola et al. 2019; Fogarty et al. 2018; Lee et al. 2019; Maher et al. 2018; Matey et al. 2020; Qin et al. 2019; Sofalvi et al. 2019). Furthermore, the method displayed acceptable precision and accuracy, combined with no ME.

Our pharmacokinetic study in rats revealed rapid increases in plasma concentrations of cyclopropylfentanyl after s.c. administration of the drug, reaching *C*_max_ by 15–27.5 min post-injection. The *T*_1/2_ of cyclopropylfentanyl in the rat was 89–115 min, which is somewhat longer than that of fentanyl (*T*_1/2_: 62–74 min; s.c.) (Liu 2009) and carfentanil (*T*_1/2_: 35–64 min; s.c.) (Bergh et al. 2019a). Present knowledge about blood concentrations of cyclopropylfentanyl after human exposure originates mainly from post-mortem case work, with reported concentrations in the range of 0.80–286 ng/mL (Bergh et al. 2018; Brede et al. 2019; Brockbals et al. 2018; Busardo et al. 2019; Danaceau et al. 2020; EMCDDA 2018a; Fagiola et al. 2019; Fogarty et al. 2018; Lee et al. 2019; Maher et al. 2018; Matey et al. 2020). Only one study has reported circulating cyclopropylfentanyl concentrations in overdose survivors, with blood concentrations of 51 and 76 ng/mL; and these subjects were also under the influence of alcohol and/or other drugs (Müller et al. 2019). The blood concentrations of cyclopropylfentanyl found in human case work overlap with the plasma concentrations in rats given s.c. injections of 30–300 μg/kg doses (see Fig. 4), suggesting that our preclinical rat model has translational value with regard to absolute blood concentrations of the parent drug. A previous study on the pharmacokinetics of the fentanyl analog carfentanil demonstrated nonlinear accumulation of the drug by comparing predicted and observed *AUC* (Bergh et al. 2019a); however, such accumulation of drug, caused by impaired clearance, was not seen for cyclopropylfentanyl. Furthermore, the observed pharmacodynamic effects were correlated with plasma cyclopropylfentanyl and its metabolite. Even though pharmacodynamic effects of cyclopropylfentanyl administration correlated with concentrations of its metabolite, it seems unlikely that cyclopropylnorfentanyl contributes to the observed effects since *N-*dealkylated fentanyl metabolites are typically inactive at μ-opioid receptors (Armenian et al. 2018).

*In vitro* studies using human liver microsomes and hepatocytes have identified the *N-*dealkylated metabolite, cyclopropylnorfentanyl, as the major metabolite of cyclopropylfentanyl (Bergh et al. 2019b; Cutler and Hudson 2019; Wallgren et al. 2020; Åstrand et al. 2018). Our preclinical study confirmed formation of cyclopropylnorfentanyl *in vivo* in rats. Busardo et al. (2019) reported cyclopropylnorfentanyl concentrations in post-mortem blood samples in the range of 5–54 ng/mL, of which the lower range corresponds to the observed *C*_max_ after injection of 300 µg/kg cyclopropylfentanyl in the rat. However, the plasma concentration of cyclopropylnorfentanyl in the rat was lower than the parent drug at all time points. This observation differs from human case studies where the metabolite is always found in higher concentrations than the parent drug after fatal intoxications (Busardo et al. 2019). This discrepancy could be a result of interspecies differences in enzyme activities (e.g., CYP3A4) in rats versus humans (Armenian et al. 2018).

In summary, we have provided novel *in vivo* pharmacokinetic data about plasma concentrations of cyclopropylfentanyl and its major metabolite cyclopropylnorfentanyl in rats, as well as new knowledge about the potency and pharmacodynamic effects of the drug. Cyclopropylfentanyl produces typical opioid-like analgesia in rats, with potency similar to the published analgesic potency of fentanyl and approximately 100 times greater than that of morphine. Plasma concentrations of cyclopropylfentanyl measured in rats receiving 30 to 300 µg/kg doses overlapped with previously reported blood concentrations of the drug after fatal intoxications in humans; however, the ratio between parent drug and metabolite are different in rats and humans, indicating possible species differences in enzyme activity. The new knowledge of the pharmacokinetics and pharmacodynamic properties of cyclopropylfentanyl presented in this study could be valuable when assessing the impact of this drug in clinical and forensic toxicology cases.
